# Social anxiety in adults with autism: a qualitative study

**DOI:** 10.1080/17482631.2020.1803669

**Published:** 2020-08-20

**Authors:** Debbie Spain, Esra Zıvralı Yarar, Francesca Happé

**Affiliations:** a Institute of Psychiatry, Psychology & Neuroscience, King’s College London, London, UK; b South London & Maudsley NHS Foundation Trust, England; c Department of Psychology, Social Sciences University of Ankara, Ankara, Turkey

**Keywords:** Autism, adults, social anxiety, social phobia, qualitative study

## Abstract

**Purpose:**

Many individuals with autism experience social anxiety (SA), yet, to date, this has almost exclusively been investigated using quantitative research methods. We know very little about why individuals with autism perceive they develop SA, what they view the impact and consequences of symptoms to be, and which coping strategies they find helpful.

**Methods:**

Using a qualitative study design, six men with autism (aged 23–52 years old) participated in individual semi-structured interviews. Data were transcribed verbatim and analysed using thematic analysis.

**Results:**

Seven overarching themes were identified: (1) causal influences for SA; (2) anxiety-provoking social situations; (3) symptoms of SA; (4) chronicity; (5) coping; (6) impact; and (7) interventions.

**Conclusions:**

Further studies are needed to more fully establish why individuals with autism are vulnerable to developing SA, to inform development of targeted interventions.

## Introduction

Autism spectrum conditions (henceforth, autism) are common neurodevelopmental conditions, affecting at least 1% of the population (Brugha et al., 2011). Core symptoms of autism include social communication impairments, restricted and repetitive interests and behaviours and hypo- and hyper-sensory sensitivities (American Psychiatric Association [APA], 2013). Autism is a heterogeneous condition; symptoms lie on a continuum, ranging from subtle through to severe. Consequently, many individuals only receive a diagnosis later in life, despite symptoms interfering substantially with functioning across the lifespan (National Institute for Health and Care Excellence [NICE], 2012).

Rates of co-occurring mental health problems are incredibly high. More than 40% of adults with autism have one, or more, mental health conditions, including, anxiety disorders, obsessive compulsive disorder (OCD), low mood and depression, psychosis and post-traumatic stress disorder (PTSD; Joshi et al., 2013; Lever & Geurts, 2016; Russell et al., 2016). Mental health comorbidities exacerbate functional impairment, reduce propensity for independent living, decrease health-related quality of life and increase carer burden (Mason et al., 2018; Murphy et al., 2018).

Social anxiety (SA; including social anxiety disorder and social phobia), in particular, commonly co-occurs with autism. For example, 13–40% of clinically referred adults with autism aged between 18 and 74 years old have been found to meet diagnostic criteria for SA when assessed by health professionals (e.g., Hofvander et al., 2009; Joshi et al., Ketelaars et al., 2008; Lugnegård et al., 2011; Roy et al., 2015; Russell et al., 2016). Rates are higher still in research samples, with 50% of adults with autism, aged up to 45 years old, scoring above suggested indicative thresholds on self-report screeners of affective, cognitive and behavioural features associated with SA (e.g., Capriola et al., 2016; Spain et al., 2016). The wide variation in SA prevalence estimates is likely to be attributable to several factors, such as impairments in introspection (e.g., alexithymia) and interoception (thereby meaning others are not made aware of anxiety symptoms), difficulties in communication between individuals with and without autism (the so-called ‘Double Empathy’ problem; Milton, 2012), diagnostic overshadowing (whereby co-occurring anxiety symptoms are ‘overshadowed’ by core autism features) and measurement issues (e.g., a lack of validated psychopathology rating scales for autism samples; Brugha et al., 2015; Kreiser & White, 2014). Nevertheless, data from multiple samples, across settings, consistently indicate that a significant proportion, but not all, individuals with autism have SA. There is an impetus, therefore, to better understand why symptoms develop, what their resultant impact and impairment is and which coping strategies individuals find more or less useful.

SA is characterized by physiological anxiety manifesting before or during social situations, concerns about negative evaluation and a tendency for avoiding interactions (APA, 2013). For some individuals, symptoms are mild, solely occurring in specific contexts (e.g., workplace presentations or at parties); for others, SA is pervasive, affecting most interactions (NICE, 2013). Symptoms typically start during adolescence, and persist for several years before help is sought. Importantly, SA is associated with an increased risk of other clinical problems, such as anxiety and affective disorders, excessive substance or alcohol use and poorer quality of life (Wong et al., 2012).

A combination of psychosocial mechanisms have consistently been found to increase susceptibility for SA symptom development in neurotypical samples. These include an inhibited temperament (Clauss & Blackford, 2012), adverse social experiences such as bullying, especially during formative years (Siegel et al., 2009), negative beliefs (e.g., about inferiority) and biases in emotion, information and attention processing (Clark, 2001). Prior to and during social situations, individuals tend to experience physiological arousal (anxiety), alongside negative automatic thoughts (e.g., about performance) and negative self-imagery (Ng et al., 2014). This usually results in avoidant and impression management coping styles (Piccirillo et al., 2016), such as avoiding situations or leaving them earlier than anticipated, mental rehearsal, staying quiet or on the edge of a group and drinking alcohol. While these coping strategies may offer short-term relief, they do not allow for disconfirmation of concerns, or negative thoughts and beliefs. Thus, they are best described as ‘safety behaviours’ (responses designed to reduce symptoms, but which indirectly serve to exacerbate these) (Clark, 2001). Encouragingly, talking therapies, specifically, cognitive therapy (CT) and cognitive behaviour therapy (CBT), are effective for treating SA, when evaluated in neurotypical samples (NICE, 2013).

Studies estimating prevalence rates of SA, alongside other mental health conditions, in individuals with autism have been relatively forthcoming. Research about contributory mechanisms and correlates for SA in the context of autism has started to emerge.

A limited number of qualitative studies have investigated anxiety in adults with autism, highlighting issues associated with SA. Trembath et al. (2012), and Halim et al. (2018), for example, explored the nature and impact of anxiety symptoms in ten adults with autism, via a series of focus groups. Both studies analysed data thematically and found that there were multiple ‘triggers’ for anxiety. These included difficulties with subtle and overt aspects of social interaction (e.g., generally being with others, using and interpreting varied forms of non-verbal communication, managing sensory sensitivities), worry about uncertainty and change to routines, and concerns about disappointment, rejection or negative evaluation. Interestingly, putative causal mechanisms appeared to relate to both core autism features (e.g., arising as a consequence of social communication impairments, sensory sensitivities and preferences for routine), as well as non-autism-related cues (e.g., concern about large social gatherings and negative beliefs about performance). Participants stated that autonomic anxiety symptoms could manifest quite quickly or build up slowly (perhaps dependent on context), and that the most common coping strategy, was to avoid situations. In a third qualitative study, Robertson et al. (2018), interviewed ten adults with autism using a semi-structured topic guide, and also analysed data thematically. Participants similarly mentioned that anxiety could occur as a result of core autism characteristics or their impact (e.g., ‘miscommunication’, difficulties tolerating change and uncertainty). Participants’ coping strategies included those implemented in anticipation of anxiety-provoking situations (e.g., establishing and adhering to a routine) and those employed in the situation (most commonly, avoidance).

Quantitative studies have also investigated SA in the context of autism, with one strand of research focusing on dimensional traits observed in non-clinical samples, and the second, focusing on traits in individuals with a confirmed clinical diagnosis of autism. Studies have almost exclusively used cross-sectional designs, with some replication of methods.

A handful of studies, for example, have recruited non-clinical university student samples (with a maximum sample size of 623 individuals), to explore relationships between autism and SA (Dickter et al., 2018; White, Bray et al., 2012; White, Kreiser et al., 2012). Participants across studies completed the Autism Quotient, that assesses behaviours and preferences suggestive of autism (AQ; Baron-Cohen et al., 2001a), and the Social Phobia Anxiety Inventory—23, that assesses behavioural, cognitive and affective symptoms indicative of SA (SPAI, 23; Roberson-Nay et al., 2007). Findings indicated that traits could co-occur and be highly correlated, yet this was not merely attributable to overlap of features across conditions or an artefact of measurement error; instead, they appeared to be separable traits. For example, Dickter et al. (2018) found that autistic traits and traits of SA correlated distinctly and differently with performance on an attentional control (flanker) task and a visual search task, both involving emotional faces.

In clinical samples, several studies recruiting adults with autism aged up to 72 years old (with a maximum sample size of 172), have examined potential associations between self-ratings of autism (with the AQ), and anxiety and avoidance associated with SA, measured with the Liebowitz Social Anxiety Scale (LSAS; Liebowitz, 1987) (e.g., Bejerot et al., 2014; Kanai et al., 2011; Lever & Geurts, 2016; Spain et al., 2016). Studies have consistently reported positive associations, whereby higher levels of autism symptomatology (i.e., more severe symptoms) are correlated with higher levels of SA symptoms (i.e., increased anxiety). Similar findings have also been described in one study looking at relationships between general social skills (e.g., assertiveness and cooperation) and autism, in an adolescent and adult sample, whereby more impaired social skills were associated with higher levels of SA (Maddox & White, 2015). Conversely, studies investigating SA in adolescents and adults with autism using other self-report questionnaires, notably the Brief Fear of Negative Evaluation Scale, that measures negative worries and beliefs associated with SA (BFNE; Leary, 1983), and the SPAI, have reported no significant associations with core traits (e.g., Capriola et al., 2016; Corden et al., 2008) (see Spain, 2018 for comprehensive review).

Research into other potential contributory mechanisms for SA, such as those highlighted above for neurotypical samples, have received far less attention in the autism literature. A small number of studies have focused on social correlates of SA in autism. In a sample of 32 individuals with autism, aged up to 45 years old, Chen et al. (2016) noted that participants experienced higher rates and levels of SA when in the company of family and friends, as opposed to when with acquaintances or strangers. Participants with anxiety and more severe autism were also found to engage more often with solitary activities. Moreover, a positive association between loneliness and SA was observed, whereby higher SA correlated with higher rates of loneliness; a finding also observed in another study of younger individuals with autism (White & Roberson-Nay, 2009). In terms of the impact of adverse social experiences, two studies of children and adolescents with autism (with a maximum sample size of 73) reported significant relationships between bullying and victimization and SA (Deckers et al., 2017; Van Schalkwyk et al., 2018). Studies focusing on these issues in adult autism samples await replication.

A handful of cross-sectional studies have investigated relationships between cognitive correlates and SA, primarily in younger individuals with autism. There is very little evidence to suggest a significant link between IQ and SA (Maddox & White, 2015; Meyer et al., 2006; Spain et al., 2016), although this has largely only been examined in individuals without an intellectual disability, rather than individuals representing the spectrum of cognitive ability. Three studies (with a maximum sample size of 163) have looked at relationships between theory of mind (ToM) and SA, none of which described significant findings (Brewer et al., 2017; Spain et al., 2016; Usher et al., 2015). One very recent study of adolescents with autism noted significant associations between intolerance of uncertainty (IoU) and SA, whereby higher levels of SA correlated with poorer IoU (Pickard et al., in press).

Finally, a few cross-sectional studies have focused on links between emotion recognition and/or regulation, and SA, primarily in younger individuals with autism. Emotion recognition has most commonly been assessed using computerized facial recognition tasks, depicting primary (basic) emotions. Findings are mixed, with some studies reporting no significant relationships between emotion recognition and SA (Meyer et al., 2006; Spain et al., 2016), and other studies noting that higher levels of SA predicted either lower fear recognition (Corden et al., 2008) or fixation duration to anger or disgust (Wong et al., 2012). Findings are more consistent with regard to associations between emotion regulation and SA. Studies examining this in moderately sized samples of adolescents and adults (with a maximum sample size of 69 individuals with autism) have found that poorer emotion regulation (i.e., emotion dysregulation) is correlated with higher levels of SA (Pickard et al., in press; Swain et al., 2015). In terms of affect, higher levels of aggression and anger (Ambler et al., 2015; Pugliese et al., 2013), and higher rates of depression (Kanai et al., 2011; Nah et al., 2019), have also been significantly linked to increased SA in individuals with autism, yet whether these develop concurrently or one precipitates the other is not established.

In summary, previous research has investigated SA in relation to sub-clinical autistic traits and in individuals with autism, with consistent evidence suggesting a significant association between core and co-morbid traits. Yet, overall, most studies have gathered information about global autism characteristics and some anxiety symptoms (e.g., physiological arousal and/or avoidance of social situations), via self-rating scales. There are obvious advantages to this approach (e.g., standardization and replication). Yet, this means there is limited facility for participants to elaborate and describe symptoms or worries in more detail. Consequently, little is known about the specific core and negative beliefs, or negative imagery, individuals with autism might experience that underpin or maintain SA. Moreover, the pooling of questionnaire items for sub-scale or total scale analyses can mean that nuanced detail is inadvertently missed. Also, we still know relatively little about the experience, impact and consequences of SA in individuals with autism or how individuals cope with this. Previous qualitative studies have indicated that general avoidance is a common coping strategy employed by individuals for dealing with anxiety, yet it is unclear whether there might be additional forms of more subtle avoidance that are employed specifically to cope with SA. Similarly, it is unclear if individuals use a broader repertoire of safety behaviours to cope with SA, as is characteristic of individuals with SA and without autism. Understanding the experience of SA—including the range of cognitive and affective symptoms that might occur, and the nature and relative success of varied coping strategies used—in this clinical population more fully, is crucial for informing the development of SA-specific evidence-based interventions (e.g., CBT), tailored for the needs of individuals with autism. To illuminate these issues more specifically, a logical next step in research is to employ qualitative methods, to find out from individuals with autism more directly about SA. In turn, this can lead to the refinement of questions, hypotheses and methodological approaches for future quantitative or qualitative research endeavours.

### Study aims

Using a qualitative study design and in-depth interviews, this study aimed to better understand the global experience of SA from the perspectives of adults with autism.

## Materials and methods

### Participants

Participants were recruited from clinical research databases hosted by King’s College London and the South London and Maudsley NHS Foundation Trust. We used a convenience sampling strategy. Study eligibility criteria were: (1) confirmed clinical diagnoses of autism (any-subtype, e.g., autism and Asperger syndrome) and social phobia or social anxiety disorder, as per ICD 10 criteria (World Health Organisation [WHO], 1992); and (2) capacity to consent to research. Exclusion criteria were: (1) IQ ≤ 69 (denoting an intellectual disability); (2) psychotic symptoms or severe depression; (3) excessive alcohol or substance use; and (4) requiring an interpreter (due to resource constraints). Of seven men approached, six agreed to participate. We did not press for reasons about non-participation. Of note, we did not intend to exclude women (see Limitations section below), yet during recruitment, we were unable to make contact with potentially eligible women. We ceased recruitment when data saturation was reached, which we defined as having taken place when no new emergent comments or concepts were mentioned by participants (Saunders et al., 2018).

### Measures

We designed a semi-structured topic guide for the in-depth individual interviews (available from the first author). This covered the following areas: types of anxiety-provoking social situations; physiological symptoms, emotions and thoughts/beliefs associated with SA; imagery; symptom modifiers; responses and coping strategies; impact; frequency and chronicity; perceived causes; and treatment. Questions were based on: (1) those typically asked during a clinical assessment (e.g., by psychiatrists and therapists), for example, “I’d like to ask you about some of the thoughts and feelings that you might experience when you feel anxious”, “I’d like to ask you about factors that seem to make the anxiety better or worse”; and (2) an interview schedule developed for a seminal qualitative study about imagery in SA in neurotypical adults (see Hackmann et al., 1998) and a revised version of this used with individuals with SA and psychosis (Lockett et al., 2012). These included questions such as, “I’m also interested in the little pictures or images people get when they are nervous. Do you ever have images like that when you are anxious either in social situations, or in anticipation of them?” and “Thinking about the image/impression, is your main impression one of viewing the situation as if looking out through your eyes, observing the details of what is going on around you, or is the main impression of observing yourself, looking at yourself from another person’s point of you?”.

Participants completed self-report questionnaires of cognitive, affective and behavioural features of SA, general anxiety and mood, either at their interview (participants could choose whether to do so before or after the actual interview had taken place) or up to 1 month before their appointment, as part of data collection for an allied study about SA in adults with autism.

The Liebowitz Social Anxiety Scale (LSAS; Liebowitz, 1987) is a 24-item questionnaire, rating anxiety about and avoidance of specific social situations (e.g., ‘telephoning in public’). Items are scored on a 3-point Likert scale. The maximum score is 144. Scores over 30 indicate possible SA and scores over 60 suggest definite SA.

The Brief Fear of Negative Evaluation Scale (BFNE; Leary, 1983) is a 12-item questionnaire, rating the degree of conviction in negative beliefs suggestive of SA (e.g., ‘I worry about what other people will think of me even when I know it doesn’t make any difference’). Items are scored on a 5-point Likert scale. The maximum score is 48. A score of 25 or more represents significant negative beliefs.

The Hospital Anxiety and Depression Scale (HADS; Zigmond & Snaith, 1983) is a 14-item questionnaire, measuring affective, behavioural and cognitive symptoms indicative of general anxiety and depression (e.g., anxiety symptoms: ‘I feel tense or wound up’ and depression symptoms: ‘I still enjoy the things I used to enjoy’). Items are scored on a 3-point Likert scale. The maximum score for each subscale is 21 and the clinical threshold is 8.

### Ethical approvals

Ethical approvals were obtained (REC REF 14 0558). Participants read the study information sheet and could ask questions prior to giving written informed consent. Five participants agreed to be audio-recorded. The sixth person declined and consented for hand-written notes to be taken. All agreed to anonymized quotes being used for dissemination.

### Procedure

Interviews were conducted by one researcher; a postdoctoral clinician with 20 years’ experience working with adults with autism, and with Master’s level training in qualitative research methods. Participants chose the time and date of their interviews, which took place at the university. Each interview started similarly: an outline of topics that would be explored and the same initial open-ended question: “I’d like to start by asking if you ever get anxious in social situations? I wonder if you could tell me a bit about this”. One participant requested a break. Audio-recording of sessions occurred for five interviews. Notes were made contemporaneously and handwritten reflections recorded after each interview.

### Data analysis

Audio recordings were listened to by the principal researcher and then transcribed verbatim. Data were analysed using thematic analysis, which comprises six stages: “(1) familiarising yourself with the data; (2) generating initial codes; (3) searching for themes; (4) reviewing the themes; (5) defining and naming themes; and (6) producing the report” (see Braun & Clarke, 2013, p. 87). DS and EZ read the transcripts independently, sentence by sentence, and highlighted initial topic areas (codes). Next, the narrative (statements) from all participants relating to each tentative code were clustered together, so as to evaluate the degree to which these appeared connected. Statements were reordered where necessary; initial thematic headings were then ascribed to sets of codes. Following this, we shared the annotated transcripts and lists of categories and codes; some of which were further annotated. Over several meetings, we developed definitions and final names for themes. During this process, we considered the ways in which our professional backgrounds (academic vs. clinical), attitudes and beliefs about social norms and conventions (being born in different parts of Europe) and conceptualizations of autism and SA (categorical vs. dimensional conditions; formulation vs. diagnosis-led approaches) might influence our views about the data and resultant codes and themes. We found, for example, that there was some divergence in our views about whether sensory sensitivities were a cause or consequence of aversive social situations. Additionally, we had different ideas about the degree to which subtle or overt avoidant safety behaviours might be useful or unhelpful in the short term vs. the longer term.

## Results

### Participant characteristics

All participants had a confirmed clinical diagnosis of autism. One person was diagnosed in their teens; the remaining five were referred for an assessment in adulthood. Diagnostic assessments of autism followed good clinical practice guidelines (NICE, 2012) and comprised the Autism Diagnostic Observation Schedule (ADOS; Lord et al., 2000), and/or Autism Diagnostic Interview—Revised (ADI-R; Lord et al., 1994), and a standard psychiatric interview. Current mental health diagnoses were confirmed via hospital records, as well as a semi-structured interview conducted in an allied study.

Descriptive analysis of questionnaire data showed that participants had high levels of affective, cognitive and behavioural characteristics associated with SA and general anxiety, and most experienced moderate levels of depression (see Table I). Five participants scored over the threshold of 60 on the LSAS, four scored over the threshold of 8 on the HADS depression subscale, and all scored over the threshold of 8 on the HADS anxiety subscale.

**Table I.** Participant characteristics.

	Mean (SD), (*n* = 6)	Range
**Age**	39.5 (12.0)	23–52
**FIQ**^1^	122.0 (23.6)	94–143
**Clinical diagnosis** *Autism* *SAD* *Depressive episode* *Dysthymia* *Low mood* *OCD* *PTSD*	6 (100%) 6 (100%) 6 (100%) 1 (17%) 2 (34%) 1 (17%) 1 (17%)	
**LSAS** *Fear* *Avoidance* *Total*	50.0 (12.3) 45.3 (14.0) 95.3 (25.4)	29–63 20-59 49–119
**BFNE**	28.2 (15.7)	10–46
**HADS** *Anxiety* *Depression*	12.7 (3.0)) 8.2 (6.1)	9–16 0-16

^1^Data were missing for one participant.

FIQ, full scale IQ; SAD, social anxiety disorder; OCD,

obsessive compulsive disorder; PTSD, post traumatic stress

disorder; LSAS, Liebowitz social anxiety scale; BFNE,

brief fear of negative evaluation scale; SBQ, social behaviours

questionnaire; HADS, hospital anxiety and

depression scale.

### Thematic analysis of qualitative data

Thematic analysis indicated there were seven overarching themes: (1) causal influences for SA; (2) anxiety-provoking social situations; (3) symptoms of SA; (4) chronicity; (5) coping; (6) impact; and (7) interventions (see Table II for an overview). Participants are assigned pseudonyms and exemplar quotes are provided.

**Table II.** Overview of themes and subthemes.

1 Causal influences • Autism characteristics Socio-communication impairments Preferences for structure and predictability Sensory sensitivities • Impairments in cognitive processing • Negative social experiences • Core beliefs • Thinking styles 2 Anxiety-provoking social situations • Being with others • Environmental cues • Social norms and conventions 3 Symptoms of SA • Physical symptoms • Negative automatic thoughts • Emotions • Imagery • Sensory symptoms 4 Chronicity 5 Coping • Coping strategies Avoidance Planning and predicting Behavioural strategies Impression management Cognitive strategies Drugs and alcohol Stimming • Usefulness of strategies 6 Impact • Social functioning • Occupational functioning • Mental health 7 Interventions • Validation of autism diagnosis • Psychological interventions CBT Mindfulness • Medication • Enhancing others’ awareness

Theme 1 Causal influences for SA

Several potential factors increasing vulnerability for SA were identified, including autism characteristics, impairments in cognitive processing, adverse social experiences and negative core beliefs and thinking styles.

Sub-theme 1.1 Autism characteristics

Three types of autism characteristics were implicated in the development of SA: socio-communication impairments; preferences for structure and predictability; and sensory sensitivities.

1.1.1 Socio-communication impairments

Participants reported that difficulties with communication and interaction had hampered their capacity to initiate and sustain fully reciprocal interactions and manage in broad ranging social situations. In turn, this increased worry about performance.

“ … I never know what to say to people … I don’t know when the person has had enough … I have noticed conversations last for variable times, and both parties seem to know when to stop and I don’t know when that is” (James)

1.1.2 Preferences for structure and predictability

Ambiguities inherent to social situations had increased participants’ apprehension about conversing with others.

“I suppose it is really down to structure, I like things to be nice and orderly and done in a particular way … say I went to a social situation and I thought, ‘okay, I have worked out that rule’. I could go to another situation that was in all aspects similar, but that rule wouldn’t be applicable that time” (James)

“Yeah, I don’t like mixed messages, you know … it’s difficult [to work things out]” (Dylan)

[A keenness for] “getting the exact right word and making sure they have understood the exact right word in the way I understand that exact word too” (Charlie)

1.1.3 Sensory sensitivities

Sensory preferences were also linked to SA. James, for example, described specific preferences when eating. He perceived others noticed his table manners, and consequently, considered him ‘different’ to them.

“I eat things all separately [on the plate] and you get a lot of people staring” (James)

Sub-theme 1.2 Impairments in cognitive processing

A consistent sub-theme concerned the impact that facets of cognitive functioning had on participants’ understanding of, and capacity to cope with, social situations. These included feeling overwhelmed, e.g., “it all builds up and what it feels like is just too much information” (James) and “less equipped to deal with frustration” (Richard). Additionally, James and Richard described a lack of social ‘intuition’ which meant they could not easily ‘read between the lines’ or anticipate others’ reactions. These difficulties had increased worry about how to interpret others’ comments and respond congruently.

Sub-theme 1.3 Negative social experiences

Participants recalled examples from childhood whereby they had been ignored or excluded by peers, leading them to feel wary of socializing.

“Others were not interested in me [at playgroup] … the other children ignored me” (Richard)

“A sense of not fitting in … a long long history of cocking things up [socially]” (Charlie)

Sub-theme 1.4 Core beliefs

Several participants described negative core beliefs, principally relating to themes of ‘difference’ and ‘inferiority’. As for individuals without autism, beliefs of this ilk partly stemmed from adverse social experiences, and exacerbated concerns about pejorative judgement.

“Like, I don’t feel I have much in common with the whole human race kind of thing … I kind of feel like I am my own independent species” (Lionel)

“I just feel I am on a lower level than, like intelligence-wise, I just feel completely inferior to people” (Dylan)

Sub-theme 1.5 Thinking styles

Alongside negative core beliefs, participants described sometimes having more negative ways of thinking. These included thinking in ‘all or nothing’ terms (Richard), a tendency for ‘paranoid thoughts’ and ‘mistrust’ about others’ actions and intentions (Lionel and Dylan). These types of thinking styles were considered to increase participants’ susceptibility for assuming others thought badly of them or intended to act negatively towards them.

Theme 2 Anxiety-provoking social situations

A range of anxiety-provoking situations were identified by participants, relating to being with others, environmental cues and social norms and conventions.

Sub-theme 2.1 Being with others

Socializing, understandably, caused anxiety. Common difficult social situations included: “meeting people I don’t know” (Charlie); ‘speaking to women’ (Dylan); ‘using the phone’ (Richard); being in ‘groups’ (Steven) or ‘crowds’ (Lionel); and dealing with ‘confrontation’ (Dylan). The number of individuals present, whether they had met previously and perceptions of how ‘friendly and non-judgmental‘ others seemed (Richard), influenced anxiety levels; anxiety increased as groups got larger and there were (more) strangers.

Sub-theme 2.2 Environmental cues

Several participants mentioned that anxiety could depend on environmental cues. ‘New places’ (Charlie), music (James), sensorially overwhelming environments such as supermarkets, pubs and clubs (Richard), places with different frequencies of ‘background noise’ (Dylan) and environments that require processing of competing sounds (e.g., others talking while the radio is on) (Charlie) were all considered anxiety-provoking. Lionel reported that “leaving the house … walking on the streets” caused anxiety, irrespective of where he was going or who with.

Sub-theme 2.3 Social norms and conventions

Adherence to social norms and conventions was deemed important by participants. Others’ failure to do this, such as being ‘late’ (James) and “not sticking to the rules” (Charlie) could contribute to a sense of ‘injustice’ (Richard) and lead participants to feel anxious about how to manage ensuing interactions. This anxiety could become exacerbated if their role within a situation was ill defined (e.g., being at a party vs. at work) (Dylan). Several participants noted that they sometimes felt conflicted, whereby they would want to behave in a certain way in social situations (e.g., listening to music while paying for shopping at the cash register), yet knew that this would contravene social conventions.

Charlie and Steven stated that their anxiety diminished when abroad (outside the UK), as they perceived that individuals elsewhere would not notice social faux pas or would forgive these.

“I have come to the conclusion that if you make a mistake abroad it is because you are English not because you are autistic … You know, that you are allowed to make mistakes, it is cultural, you are allowed to want to go off and be with your own kind for a night and speak English for a night or – you know, it is easier to find excuses” (Charlie)

Theme 3 Symptoms of SA

Symptoms of SA included physical symptoms, negative automatic thoughts, negative emotions, imagery and sensory overload.

3.1 Physical symptoms

All participants described a range of physiological symptoms indicative of anxiety, occurring either before or during social situations.

“My stomach starts churning away … I spent the whole night before being sick … I do get headaches, but I presume that is because my muscles are sort of tensed up and it then transfers itself to my head” (James)

“Increased heart rate, a bit shaky … a bit fidgety … I notice I am sort of salivating a bit more” (Steven)

Dylan reported feelings suggestive of dissociation.

“I can be in a room full of people and feel solo, you know? Yeah, everything is going on and I just feel like I am standing there, you know? Just like I am in a cloud, do you know, like I am in a bubble?” (Dylan)

3.2 Negative automatic thoughts

In discussing their thinking about social interactions, participants listed (negative) automatic thoughts relating to ‘knowing what to say … how to respond’ and worries about negative evaluation.

“I suppose there is a slight fear you might say the wrong thing or you try and make a joke out of something and it might be slightly inappropriate or rude … I assume others will judge me for not working” (Charlie)

“Does that person like me?” (Richard)

3.3 Emotions

Several negative emotions were reported to manifest alongside anxiety. Lionel and Steven mentioned feelings of anger and annoyance (at themselves or others), upset and disappointment. Charlie stated he could become embarrassed (e.g., if he noticed others noticing him). Lionel and Richard described feeling vulnerable. Negative emotions primarily occurred during or after social interactions—alongside physical symptoms outlined above—especially when with unfamiliar others or in unstructured situations.

3.4 Imagery

When asked about imagery associated with SA, two participants stated they have a visual impression of how others perceive them; as though they were adopting an observer perspective.

“[I am] not very confident, I think I start mumbling. I think I start mumbling and my head would go down, I suppose general body language you know … probably generally just look nervous I would have thought” (Dylan)

3.5 Sensory symptoms

Three participants had heightened sensory sensitivities when anxious.

“Smells, sounds they all become more intense. My sense of smell gets a lot more—it improves a lot more—I have a greater range of what I can smell and smells get stronger and sounds become stronger … Sometimes I can feel my ears widening to be able to hear more. That—that—that is a weird feeling like my ears are getting bigger and all my blood is pumping through them, I don’t understand it” (Lionel)

Theme 4 Chronicity

Participants reported that they had experienced symptoms for a substantial period.

“As long as I can remember really” (Lionel)

“ … with hindsight, clearly, I had had these issues for, well, life I suppose” (Charlie)

Theme 5 Coping

Participants reported they had different ways of coping with SA ‘in the moment’, as well as more generally, and there were two sub-themes relating to this: coping strategies and usefulness of strategies.

Sub-theme 5.1 Coping strategies

Seven types of coping strategy were identified: avoidance; planning and predicting; behavioural strategies; impression management; cognitive strategies; drugs and alcohol; and stimming.

5.1.1 Avoidance

A common response to anxiety—for individuals with and without autism—is to avoid provocative cues. Participants in the present study reported engaging in overt and subtle avoidance.

“Most things I try to shy away from” (Dylan)

“So, I would always take lunch at noon, erm, so that anywhere I was going to get lunch would be the least crowded possible at the most reasonable time for my tummy. And if I was out and about rather than office based and I missed that timeslot I would not eat” (Charlie)

5.1.2 Planning and predicting

Participants identified strategies they employed before social situations due to anticipatory anxiety. Lionel and Steven described ‘playing out’ interactions, whereby they imagined what would happen in an ensuing social event. Building on this, most participants said they tried to predict what others would say so they could plan their response. More practically, James and Lionel said they plan travel routes in advance, arrive very early to appointments and have backup travel options in case there are transport problems.

5.1.3 Behavioural strategies

Participants listed examples of behavioural strategies they had developed to cope with SA ‘in the moment’. These included: listening to music or simply wearing earphones when in public (Lionel); taking ‘props’, such as lists of conversation starters or items associated with hobbies (Steven); being selective about who to sit with, where and for how long (Steven, Lionel and James); smoking (Charlie); going for a walk (James); ensuring they had a clearly defined role (Richard); asking others to tell them when they should stop talking (James); and being assertive at work (Dylan).

5.1.4 Impression management

Some participants used impression management strategies, defined as “behaviours intended to tightly control one’s impression on others” (Piccirillo et al., 2016, p. 677), to try and ensure they came across well. Commonly, this included self-monitoring (their behaviour) and camouflaging.

“Checking whether I’m getting a positive vibe from others … looking out for this” (Richard)

“And literally, physically wearing a mask … I am consciously not doing some of the stimming things that I would do” (Charlie)

5.1.5 Cognitive strategies

Several participants described hypervigilance during social situations.

“Yeah and then I start watching the second hand and count the seconds … what sometimes happens is that I start fixating on time” (Dylan)

Additionally, engaging in post-mortem rumination was common.

“I replay the situations and kind of see how I did or what I could have done differently. It is more like listening to a recording. [Replay it forever]. It depends how good I did in the conversation—If I couldn’t think of a better response” (Steven)

5.1.6 Drugs and alcohol

Illicit substances or alcohol were used by some to manage SA symptoms. Lionel said that smoking cannabis could help him feel relaxed and better able to “deal with people”. Charlie and Dylan stated they ‘self-medicated’ with alcohol to make social situations more tolerable.

5.1.7 Stimming

One participant said that he used stimming (repetitious movements), to reduce anxiety.

“Well, I suppose actually I might do some quiet private stimming. You might sort of grab a moment to do that or pop to the loo and find yourself doing that” (Charlie)

Sub-theme 5.2 Usefulness of strategies

Importantly, participants noticed that some coping strategies were partially useful, yet did not necessarily get rid of SA. Charlie and Dylan, for example, found that drinking alcohol made them feel low. Additionally, avoidance appeared to perpetuate isolation and anxiety.

“So, it was like obviously it was like avoidance that sort of helped the actual thing [the specific event] but not the global problem” (Steven)

Similarly, post-mortem rumination served to exacerbate worries.

“ … I don’t think it is too good for me sitting in and then you start thinking and dwelling on things and the way my mind is set up; I dwell on bad things, not good things” (Dylan)

Theme 6 Impact

SA impacted on social and occupational functioning and mental health.

Sub-theme 6.1 Social functioning

All participants had relatively small social networks; smaller than they would have liked.

“ … I don’t go outside my circle of mates. I stay in my circle of friends and that is it … I have never really spoken to new people … I think my circle of friends are all the same” (Dylan)

Sub-theme 6.2 Occupational functioning

Three participants were unemployed; partly attributed to social interaction difficulties. The three participants employed described using strategies to limit the amount, range and/or unpredictability of requisite work social situations (e.g., refusing to do tasks beyond their role, declining work nights out and informing clients or employers about their general difficulties).

Sub-theme 6.3 Mental health

SA co-occurred with other clinically diagnosed mental health conditions, notably, depression and post-traumatic stress disorder (PTSD), and disrupted sleep.

“Reliving, not remembering … I am re-experiencing the same anxieties [every time I am in a social situation], it’s PTSD” (Charlie)

Theme 7 Interventions

Four distinct forms of helpful intervention were identified for addressing SA and associated negative thoughts and beliefs: validation of autism diagnosis; psychological interventions; medication; and enhancing others’ awareness.

Sub-theme 7.1 Validation of autism diagnosis

Receiving a diagnosis of autism was deemed beneficial for several reasons. First, this helped to explain why participants had experienced social difficulties early on, and thereby, facilitated the process of acceptance (e.g., of vulnerabilities).

“But then when I had my [autism] assessment, I realised there are reasons for the way I am, and I started trying to accept it more” (James)

Second, receiving an autism diagnosis had provided a springboard from which to be able to request adaptations to social situations.

“And I have learnt, you know, post diagnosis that I am starting to go to events because I can tell them my accessibility needs in advance, which is helpful” (Charlie)

Sub-theme 7.2 Psychological interventions

Participants described their experiences of psychological interventions, namely, CBT and mindfulness.

7.2.1 CBT

Five participants had been offered individual CBT for SA with clinicians who worked or had worked at autism-specialist services. Several aspects of CBT were considered useful for enhancing emotion awareness and reducing SA symptoms.

“They are sort of like flow diagrams [formulations] about situations and what I think and what to do … they help” (Steven)

“I have learnt that it is my interpretation of it” (Lionel)

Indirectly, participants highlighted the importance of a therapeutic relationship.

“It is just having someone to talk to and someone not really judging you by your answers. Just listening and giving you helpful solutions … not trying to pressure you into anything that you don’t want to do … suggestions that I get kind of from her and that she helps me come up with, I implement them in real life scenarios and they work” (Lionel)

7.2.2 Mindfulness

For two participants, mindfulness approaches helped to reduce high levels of general anxiety.

“Yeah, take a moment, take a breath” (Dylan)

Sub-theme 7.3 Medication

Three participants were prescribed antidepressants, and one a betablocker. Antidepressants were said to have improved general mood, but not SA specifically.

Sub-theme 7.4 Enhancing others’ awareness

Charlie, Dylan and Richard deemed it important for others to be more autism-aware, so that contexts could be suitably adapted and their responses better understood.

“People with autism want access to the community and coordinators [health and social care providers] need to be flexible … different needs and abilities for people with autism” (Richard)

## Discussion

Many individuals with autism experience SA. Using a qualitative study design, we aimed, for the first time, to explore the perspectives of adults with autism about the causes, symptoms, maintaining factors and consequences of SA. Participants were forthcoming during their interviews, resulting in a rich dataset.

### Psychosocial mechanisms for SA in autism

Analysis of the data suggests that a combination of psycho-social mechanisms increases vulnerability for SA in this group. Many of these, including adverse social experiences, negative core beliefs and some cognitive processes (e.g., difficulties gauging others’ intentions) are risk factors for SA in neurotypical adults (Clark, 2001). Our findings also highlight the potential contribution of core autism characteristics, and their impact, to SA development, reflecting previous general qualitative (Halim et al., 2018; Robertson et al., 2018; Trembath et al., 2012) and SA-specific quantitative research (Bejerot et al., 2014; Kanai et al., 2011; Lever & Geurts, 2016; Spain et al., 2016).

Participants described that ‘not knowing’ about the autism diagnosis until adulthood exacerbated uncertainty and worry about being different or inferior to others, and contributed to negative core beliefs; risk factors for SA. While previous studies have reported correlations between self-ratings of global autism characteristics and SA, participants in the present study described links between specific autism features and the resultant anxiety-provoking nature of social contexts. For example, difficulties with, and thus concerns about, initiating and sustaining reciprocal social interactions were said to arise in conjunction with social interactions; sensory sensitivities appeared to arise in response to aspects of the social environment; and preferences for structure and predictability were linked to both interactions and environments. This is important as it implies that SA may develop in individuals with autism, in part, due to environmental cues that are not social, which is generally uncharacteristic of non-autistic individuals with SA.

It is beyond the scope of our dataset to assert which psycho-social mechanisms are most pivotal; there are clearly distinct types of mechanisms (e.g., autism features, adverse experiences and so forth), yet the degree to which these are salient likely varies from person to person. This may also depend on systemic considerations (e.g., whether the family are sociable or reserved, how parents help their children to cope), and protective factors (e.g., positive friendships). Further longitudinal studies are needed to investigate causal mechanisms for SA in autism more fully. Ideally, studies should recruit individuals across the lifespan, with and without ID, to clarify if risk factors differ, for example, according to gender, ID or age.

### SA symptomatology

Previous cross-sectional (quantitative) studies have found that individuals with autism endorse cognitive, affective and behavioural characteristics associated with SA. However, use of a qualitative approach here allowed us to ask about these more openly and broadly, resulting in the description of a range of SA symptoms and experiences.

Heightening of senses was mentioned by several participants. In neurotypical adults, it has been hypothesized that sensory sensitivity is a risk factor for SA, although data are not wholly consistent (see Hofmann & Bitran, 2007). In relation to research with individuals with autism, our findings reflect those from a qualitative study conducted with adults with Asperger syndrome (Smith & Sharp, 2013), who reported they commonly experienced heightened senses, which coincided with stressful events and, in turn, could further exacerbate stress. Moreover, these findings tally with those of previous qualitative studies, in which adult participants have noted sensory sensitivities to coincide with general anxiety (Halim et al., 2018; Trembath et al., 2012). Whether these are a cause or consequence, or both, of SA, is less clear. Studies employing longitudinal designs may shed more light on this issue.

Two participants reported self-focused negative imagery: in their ‘mind’s eye’, they imagined others could see flaws in their appearance and behaviour. Negative imagery is a hallmark characteristic of SA in neurotypical individuals and a central tenet of prevailing CBT conceptualizations and treatments (Clark, 2001). Imagery has seldom been explored in individuals with autism, and not at all in SA, yet a seminal study by Ozsivadjian et al. (2017) found that children and adolescents with autism and high (general) anxiety had more spontaneous images than highly anxious children without autism. Further, characteristics such as imagery ‘vividness, controllability and realism’ did not differ significantly between autism and non-autism groups. It remains to be seen whether adults with autism show the same frequency and quality of imagery as neurotypical adults, and if these are associated with SA or other mental health conditions.

Taken together, future studies should establish the range and severity of SA symptoms experienced by individuals with autism, potentially combining mixed methods approaches. Given the consistently strong relationships between general anxiety and sensory under- and over-responsivity in individuals with autism (see South & Rodgers, 2017), the degree to which sensory processing is a symptom and/or predisposing mechanism for SA should also be investigated.

### Coping strategies for SA

Participants identified a range of coping strategies, employed before, during and after anxiety-provoking situations. Specific coping strategies, notably avoidance, are commonly observed in individuals with anxiety disorders (Helbig‐Lang & Petermann, 2010). Moreover, avoidance has been described as the prevailing coping strategy by adults with autism and general anxiety (Halim et al., 2018; Robertson et al., 2018; Trembath et al., 2012). Other coping strategies, such as those relating to impression management and cognitive strategies are primarily seen in neurotypical adults with SA, as the underlying intention is to reduce the likelihood of negative evaluation. Yet, as described in the wider neurotypical SA literature, some coping strategies indirectly reinforce, i.e., maintain anxiety over time, such as monitoring performance during interactions. Our findings suggest, for the first time, that there are some commonalities in coping strategies, and thus, potentially maintaining mechanisms, for individuals with SA with and without autism, and this warrants further research.

Participants gave examples of two autism-specific coping strategies, namely, camouflaging and stimming. There has been burgeoning interest in the concept of camouflaging. Data from quantitative (Lai et al., 2017) and qualitative (Hull et al., 2017) studies indicate that adults may camouflage ‘their autism’ for reasons including avoiding bullying, trying to fit in and wanting to increase their social network. It is unclear whether attempts to camouflage by study participants here occurred to a greater (more frequent) extent than in adults with autism without SA, and whether the drivers for this were related to concerns about negative evaluation. It would be useful for research in this area to compare reasons for, and behaviours associated with, camouflaging in individuals with autism, with no and/or varied mental health conditions.

### Interventions for SA

Finally, participants described several interventions they found beneficial. Different elements of CBT were deemed useful, including enhancing awareness of emotion recognition (and subsequently, regulation of this), identifying and challenging negative thoughts, and finding new ways of understanding, coping with and responding to anxiety-provoking cues. A recent systematic review highlighted the potential utility of CBT for SA in autism (Spain et al., 2017), although there is a dearth of rigorously conducted studies. Thus, empirical development and evaluation of psychological interventions is an important research priority.

### Limitations

There are several study limitations. Although unplanned, our sample solely comprised men, despite concerted efforts to recruit women, including liaising with clinicians and research collaborators. Most participants were Caucasian, which was also unintentional. Our findings, therefore, may not be reflective of the experiences of women (who may be more likely to camouflage than males, and thus have different social experiences, concerns or anxiety to males; Lai et al., 2017), individuals from BAME backgrounds, or individuals who have a concurrent ID. Additionally, all bar one participant were diagnosed with autism in adulthood, and so we were unable to examine the degree to which earlier vs. later diagnosis might have influenced development of core beliefs, coping styles, social experiences or SA. We did not assess neuropsychological functioning, yet it could have been informative to measure ToM, attention or information processing, to establish whether any of these facets were related to SA or low mood. The study design and methods stemmed from discussions with adults with autism in clinical and research settings. Yet, we acknowledge that these could have been further informed by service user consultation and collaboration. Finally, we did not recruit adults with autism without SA, and so it was not possible to compare and contrast themes about social interaction across groups to establish what might be specific to autism plus SA.

### Conclusions

SA occurs commonly. Findings here indicate that SA in adults with autism is associated with the range of cognitive and affective features, and behavioural responses, frequently observed in individuals with SA without autism. Moreover, data suggest that individuals with autism may develop SA for comparable reasons to individuals without autism (e.g., adverse social experiences), as well as mechanisms that may be unique to this clinical population (e.g., arising as a consequence of core traits). Cross-sectional and longitudinal mixed methods research is needed to better understand SA in individuals with autism, so as to inform treatment approaches.
